# The Emergence of a Sustainable Tobacco Treatment Program across the Cancer Care Continuum: A Systems Approach for Implementation at the University of California Davis Comprehensive Cancer Center

**DOI:** 10.3390/ijerph17093241

**Published:** 2020-05-06

**Authors:** Elisa K. Tong, Terri Wolf, David T. Cooke, Nathan Fairman, Moon S. Chen

**Affiliations:** 1 Department of Internal Medicine, University of California, Davis, Sacramento, CA 95817, USA; 2 Comprehensive Cancer Center, University of California, Davis, Sacramento, CA 95817, USA; tpwolf@ucdavis.edu (T.W.); npfairman@ucdavis.edu (N.F.); mschenjr@ucdavis.edu (M.S.C.J.); 3 Department of Surgery, University of California, Davis, Sacramento, CA 95817, USA; dtcooke@ucdavis.edu

**Keywords:** tobacco cessation, cancer care continuum, implementation research

## Abstract

Tobacco treatment is increasingly recognized as important to cancer care, but few cancer centers have implemented sustainable tobacco treatment programs. The University of California Davis Comprehensive Cancer Center (UCD CCC) was funded to integrate tobacco treatment into cancer care. Lessons learned from the UCD CCC are illustrated across a systems framework with the Cancer Care Continuum and by applying constructs from the Consolidated Framework for Implementation Research. Findings demonstrate different motivational drivers for the cancer center and the broader health system. Implementation readiness across the domains of the Cancer Care Continuum with clinical entities was more mature in the Prevention domain, but Screening, Diagnosis, Treatment, and Survivorship domains demonstrated less implementation readiness despite leadership engagement. Over a two-year implementation process, the UCD CCC focused on enhancing information and knowledge sharing within the treatment domain with the support of the cancer committee infrastructure, while identifying available resources and adapting workflows for various cancer care service lines. The UCD CCC findings, while it may not be generalizable to all cancer centers, demonstrate the application of conceptual frameworks to accelerate implementation for a sustainable tobacco treatment program. Key common elements that may be shared across oncology settings include a state quitline for an adaptable intervention, cancer committees for outer/inner setting infrastructure, tobacco quality metrics for data reporting, and non-physician staff for integrated services.

## 1. Introduction

Tobacco treatment is increasingly recognized as important to prevent cancer, and comprehensive tobacco treatment in the oncologic setting can help patients quit [1]. Tobacco use is the principal risk factor for at least 12 cancer types and for the leading cause of cancer mortality (lung) [2]. The use of tobacco products has immediate cardiopulmonary, immune, and metabolic effects that can worsen cancer treatment outcomes (e.g., surgery [3]), increase side effects, and raise recurrence rates and mortality [2]. However, a 2013 study of tobacco treatment services at National Cancer Institute (NCI) Comprehensive Cancer Centers showed a dearth of programs dedicated to helping cancer patients stop using tobacco products [4]. There is a demonstrated need for implementation of tobacco treatment programs in oncology settings and with cancer providers [5,6] and pragmatic strategies are needed [7].

In 2017, the NCI launched a Cancer Center Cessation Initiative (C3I) to competitively fund select NCI Comprehensive Cancer Centers to integrate tobacco treatment services into cancer care, funded by the NCI Moonshot program [8]. The NCI supported the time and effort of teams to plan, implement, evaluate, and sustain a comprehensive tobacco cessation program for patients with cancer, including finding ways to determine and then address the variety of implementation challenges. Each cancer center could propose their own implementation activities and funding to create a plan for building infrastructure and continue the program past the funding period.

As most cancer centers are matrix cancer centers, that is, centers embedded within a larger health system, a systems approach can develop a sustainable tobacco treatment program. Sustainability is key to addresses maintenance of an intervention while considering associated costs. As tobacco treatment itself does not generate significant revenues, it is important to understand how cancer centers and health systems are influenced by accrediting or regulatory organizations. It is also important to understand how primary care, the traditional hub for tobacco treatment, intersects with cancer care.

The purpose of this study is to describe the emergence of a sustainable tobacco treatment program across cancer care, using a systems approach at the University of California Davis Comprehensive Cancer Center (UCD CCC). For years, UC Davis has had health education group classes for tobacco treatment and in 2013 established the first electronic referral in California to its state quitline [9]. However, these tobacco treatment services had been targeted towards primary care and the hospital, and not specialty care for patients with cancer. In 2017, the NCI funded the UCD CCC in the first of two cohorts to participate in the two-year NCI C3I. Lessons learned from the UCD CCC in establishing a sustainable tobacco treatment program are illustrated across a systems framework of cancer care and by applying constructs from an implementation research framework.

## 2. Materials and Methods

### 2.1. UCD Stakeholders Involved in Implementation and Evaluation

The authors reflect the multidisciplinary team of UCD CCC stakeholders involved with ongoing discussions to develop and continuously refine a sustainable tobacco treatment program across cancer care. Initially, an institutional commitment to the NCI for sustaining a tobacco treatment program in cancer care was secured through an agreement by the UCD CCC Director and the Health System’s Chief Quality Officer. The UCD CCC tobacco treatment program activities were developed and refined by the NCI C3I project team.

The first author (E.K.T.), a general internist, led the NCI C3I project team at the UCD CCC and is also a tobacco/cancer control and health services researcher. [10] A nurse program manager (T.W.) conducted quality improvement efforts with cancer center staff and has experience as a UCD CCC nurse and in quality improvement. Senior cancer center leadership on the team includes the Associate Director for Population Sciences and Community Outreach/Engagement (M.S.C.). The NCI C3I project was placed within the UCD CCC Office of Community Outreach and Engagement as an essential part of its mission to mitigate the cancer burden and high tobacco rates for the cancer center catchment area. The Office of Community Outreach and Engagement has a community advisory board and expert stakeholder committee that regularly provide feedback for ongoing projects. In recognition of the importance of mitigating the cancer burden, the community advisory board chose tobacco control as its most actionable priority.

Additional UCD CCC clinical leaders represented here as co-authors include: the General Thoracic Surgeon (D.T.C.) who leads the Comprehensive Lung Cancer Screening (LCS) Program and the Director of Supportive Oncology and Survivorship (N.F.). Here, they represent UCD CCC activities across the Cancer Care Continuum for the domains of Screening, Diagnosis, Treatment, and Survivorship. The LCS program is represented here to exemplify Screening because it is the only tobacco-related cancer screening where the Centers for Medicare and Medicaid Services (CMS) requires tobacco treatment as part of the screening workflow.

The UCD CCC Cancer Committee was an integral forum to develop and support the tobacco treatment program as a systemwide activity. Chaired by the Physician-in-Chief for Oncology Services, the cancer committee members include physicians from each diagnostic and treatment service, and non-physician representatives from administration, coordinators, cancer registry, clinical and supportive services covering the continuum of cancer care and services. A community stakeholder from the American Cancer Society is also on the committee. Two of the authors (E.K.T. and N.F.) have been physician members of the committee for years.

### 2.2. The Cancer Care Continuum as a Systems Framework

**Figure 1:**
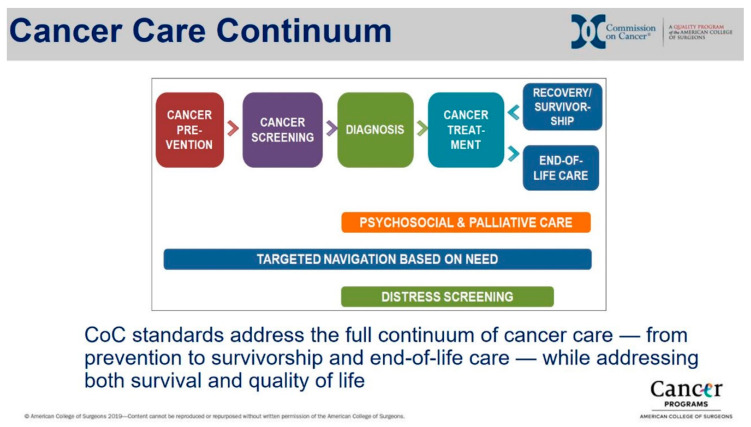
Cancer Care Continuum Domains. Permission to reprint from the American College of Surgeons’ Commission on Cancer (CoC).

Figure 1 shows the Cancer Care Continuum [11], which we adapted for this study as a systems framework. According to the NCI, the Cancer Control Continuum “is a useful framework on which to view plans, progress, and priorities. It helps us identify research gaps, where we must collaborate with others to have an impact, and where more resources may be needed.” The Continuum identifies the multiple touchpoints by providers as patients move from primary care to cancer care, with concurrent psychosocial and supportive care that continues survivorship or through the end of life, depending on the patient’s needs. Patients with recurrences or new cancers may cycle back through the Cancer Care Continuum, reflecting how the continuum is not necessarily a linear progression. Each domain is described below with regards to tobacco treatment integration.

The Prevention domain encompasses activities to prevent tobacco-related disease, typically in the primary care setting where tobacco treatment has historically developed and has the most data about its provision [12]. These activities may extend to other settings, including the hospital, specialty care, or the community, especially if a patient has not engaged with primary care. Matrix cancer centers interface with primary care and other services internal or external to their health system.

The Screening domain refers to cancer screening and detection efforts, within primary care but may extend to other settings. Cancer screening represents an opportune time to address tobacco use, and four of the 12 tobacco-related cancers (lung, colorectal, cervical, liver) have screening recommendations. However, only LCS has a mandate to provide tobacco treatment. LCS is still relatively new, with the first evidence of a mortality benefit demonstrated within the past 10 years [13,14], and, despite Medicare coverage, nationwide LCS has low utilization [15] and, as a result, low rates of provision of tobacco treatment to date.

The Diagnosis domain refers to the identification of a patient with cancer, bridging the settings of primary care, hospitals, or cancer clinics. A new cancer diagnosis and the advice of the cancer provider can be associated with being five times more likely to quit than smokers in the general population [16]. However, patients requiring supportive care may report very severe distress, such as pain, fatigue, sleep, and anxiety [17]; patients who use tobacco as a coping mechanism may have difficulty quitting. A clinical pathway for tobacco treatment for new cancer center patients has been successful at a stand-alone cancer center [18], but more data is needed including matrix cancer centers.

The Treatment domain refers to the multidisciplinary cancer care services in the cancer clinic or hospital, which can be complex in a matrix cancer center. The clinic space in the cancer center may be utilized by different departments, including medical, surgical, and radiation oncology. These departments may have different leadership and reporting structures within the health system but collaborate on cancer service lines. As a patient with cancer may have multiple providers, tobacco treatment may not have clear ownership and responsibilities across clinical services. As above, tobacco treatment has historically not been well-integrated into cancer center clinical care [4].

The Survivorship domain (which includes palliative care, not discussed here) encompasses the interprofessional and psychosocial support of supportive oncology throughout cancer care services. The interprofessional staff includes social workers, dietitians, nurse navigators and program managers serving both pediatric and adult oncology patients. For patients, survivorship can be a stressful time because of the uncertainty about recurrence, treatment side effects, the anxiety of life after completing treatment, and fewer encounters. A nationwide study of survivors [19] found that many were challenged to adhere to healthy lifestyle behaviors. Patients who quit tobacco during cancer treatment may be vulnerable to relapse. More data is needed about the provision of tobacco treatment in this growing area of Survivorship either in primary or cancer care settings [20].

### 2.3. Constructs from an Implementation Research Framework

To describe implementation, this study is organized around five constructs from the Consolidated Framework for Implementation Research (CIFR) [21,22], a pragmatic framework for which researchers can select constructs to guide diagnostic assessments of implementation context, evaluate implementation progress, and help to explain findings. The first construct of CFIR reflects the intervention: its characteristics, core components, and adaptable elements. The second construct of CFIR describes the individuals and stakeholders involved with the intervention and implementation, which is described above in Section 2.1.

The third construct of CFIR is described as the “Outer Setting”, which includes “the economic, political, and social context within which an organization resides [22].” Here, Outer Setting describes the motivational drivers (e.g., quality metrics, accreditation) at the health system level for integrating tobacco treatment throughout the Cancer Care Continuum. For the health system level, we include the primary care, hospital, and cancer specialty care settings.

The fourth construct of CFIR is called the “Inner Setting”, which includes “features of structural, political, and cultural contexts through which the implementation process will proceed.” Here, Inner Setting describes the context of clinical entities (e.g., clinics, departments) for varying levels of implementation readiness. Three subconstructs of “implementation readiness [22]” are as follows: 4a) Leadership Engagement: commitment, involvement, accountability of leaders and managers, 4b) Available Resources: the level of resources dedicated for implementation and ongoing operations including money, training, education, physical space, and time, 4c) Access to Information and Knowledge: the ease of access to digestible information and knowledge about the intervention and how to incorporate it into work tasks (i.e., workflow), and information and knowledge, which include all sources, such as experts, other experienced staff, training, documentation, and computerized information systems (i.e., information technology, training). Table 1 describes these “implementation readiness” subconstructs and the barriers and facilitators at the UCD CCC to implement tobacco treatment across the Cancer Care Continuum domains.

The fifth construct of CFIR is the implementation process. The implementation process reflects “the active change process to achieve individual and organizational level use of the intervention as designed, which may not necessarily be formally planned or linear [22].” Here, we focus on the NCI C3I project activities in the Treatment domain and how it interacted with the Prevention domain to mitigate barriers and facilitate implementation readiness.

### 2.4. Analysis

The identified barriers and facilitators across the CFIR’s three implementation readiness subconstructs and the Cancer Care Continuum (Table 1) were generated by the first two co-authors, who had actively engaged with the clinical and leadership stakeholders in Section 2.1. Additional barriers and facilitators were solicited amongst the additional co-authors. The barriers and facilitators in Table 1 are meant to highlight the UCD experience rather than be a comprehensive listing of all potential factors. Using an iterative process [23], the two co-authors reviewed the identified barriers and facilitators by the key factors addressed within each of the three implementation readiness subconstructs. The identified barriers and facilitators were further iteratively refined by discussion and consensus across the co-authors.

## 3. Results

The CFIR constructs below describe the implementation and emergence of a sustainable tobacco treatment program at the UCD CCC. The characteristics of the tobacco treatment intervention were adaptable for various settings. The outer setting of motivational drivers for the health system has positioned tobacco treatment as a priority for leadership engagement. The inner setting across the five domains of the Cancer Care Continuum shows that implementation readiness with clinical entities was higher in the Prevention domain, but Screening, Diagnosis, Treatment, and Survivorship domains demonstrated less implementation readiness despite leadership engagement. The implementation process for the NCI C3I project activities in the Treatment domain mitigated barriers and facilitated implementation readiness by enhancing information and knowledge sharing through the Cancer Committee infrastructure, while identifying available resources from the Prevention domain and adapting workflows for cancer care.

### 3.1. Characteristics of the Intervention: Referral to Quitline or UCD Group Class

Besides provider advice, the core components of the tobacco treatment program available throughout the UCD health system include 1) the Health Management and Education (HME) group class, available as an 8-class series or a 2-hour workshop, and taught by a part-time nurse certified as a tobacco treatment specialist and, with broader reach, 2) the state quitline which offers free telephone counseling services in Spanish and Asian languages. In the hospital, a nursing order for smoking cessation education would be activated on admission, and these referrals could be ordered at discharge. Tobacco treatment medications are available through physician order sets; pharmacists in California can furnish nicotine replacement therapy but this has not been adopted widely. In the hospital, pharmacy students assist some medicine teams with tobacco treatment.

The effectiveness of the quitline at UCD is similar to published literature: the 6−12 month point-prevalence smoking cessation rate for 126 patients was 21.9% and, more conservatively, 12.2% among 576 patients with a quitline referral (patients with missing data were assumed to be smoking) [9]. The effectiveness of the quitline was even higher among UCD thoracic surgery patients (*n* = 111) with 6-month point-prevalence smoking cessation rates between 24%−50%, with the highest rates among patients who were having an operation and engaged with the quitline [24].

The existing tobacco treatment program has adaptable components for cancer care. The in-person group class is open to any UCD patient in the Sacramento area. The quitline referral and order sets are available to all UCD physicians, and the quitline can service patients anywhere in the state. Medications may be picked up at the cancer center’s outpatient pharmacy.

### 3.2. Outer Setting: Motivational Drivers for the Health System

The motivational drivers at a systems level for integrating tobacco treatment into cancer care are quality programs and accreditation standards that support operations. For the health system, the quality programs may be required by various payors or accreditation programs. For the UCD CCC, the American College of Surgeons’ Commission on Cancer (CoC) and the National Cancer Institute provide accreditation standards and program expectations.

There have been several quality programs [25] that have helped the UCD health system to prioritize tobacco assessment and treatment before the NCI C3I award. As the health system adopted an electronic health record, tobacco assessment was a Meaningful Use requirement in the outpatient setting. For certification as a Primary Care Medical Home, outpatient clinics needed to report on a quality metric for tobacco assessment and treatment. For a Medicaid 1115 waiver, state public hospitals were required to report on a quality metric for tobacco assessment and treatment in order to receive substantial incentive payments; this outpatient quality metric also encompasses specialty care, but cancer clinics report to the hospital instead of the ambulatory network. While there is a hospital-based quality metric for tobacco treatment with the Joint Commission, this is a voluntary metric that has not yet been adopted. Value-based payment programs include tobacco in the available selection of elective quality metrics, for future consideration.

In 2018, in conjunction with the NCI C3I award, the UCD Cancer Committee endorsed its annual programmatic goal as tobacco assessment and treatment. The Cancer Committee, described above, is a program requirement by the CoC [26], a quality program that accredits hospitals, freestanding centers, and integrated cancer networks. The supportive infrastructure allowed the NCI C3I project team to embed implementation formally and informally into the cancer center clinical operations and provide a reporting and dissemination mechanism. Dissemination of the program development and progress occurred through formal reports and informal discussions at or outside bi-monthly meetings, as well as meetings with clinical leadership and departments.

As part of the NCI C3I award implementation, the C3I project was enthusiastically embraced as essential and placed as part of the UCD CCC’s Office of Community Outreach and Engagement (COE). In the most current Program Announcement (PAR−20−043) governing the evaluation of NCI cancer centers, mitigation of the cancer burden for a cancer center’s catchment area is an expected responsibility, particularly for assessing COE. This placement assures both an intended emphasis on patients with cancer in both in-patient and out-patient settings as well as outreach to patients’ families and the population at-large. This is particularly important since the catchment area’s tobacco use rates exceed the state’s and mitigating the impact of tobacco is the motivating force for enhancing cancer treatment outcomes, survivorship, and overall population health.

### 3.3. Inner Setting: Implementation Readiness across the Domains of the Cancer Care Continuum

Table 1 demonstrates three subconstructs of implementation readiness for tobacco treatment, with its barriers and facilitators, across five Cancer Care Continuum domains at the UCD CCC. While the Prevention domain already had established tobacco treatment workflows, the Screening, Diagnosis, Treatment, and Survivorship domains demonstrated less implementation readiness despite Leadership Engagement. The Screening and Survivorship domains both have growing programs with potential for implementation readiness with Available Resources and Information and Knowledge Sharing with the tobacco treatment program staff. The Diagnosis domain has lower implementation readiness across the three subconstructs. The Treatment domain initially had low implementation readiness due to challenges with Available Resources and Information and Knowledge Sharing, even though similar clinic workflow processes existed in the Prevention domain.

#### 3.3.1. Prevention: Primary Care and Other Settings

The strong history of Leadership Engagement for improving tobacco treatment implementation at the UCD health system since 2012 has led to established Available Resources and Access to Information and Knowledge workflows for primary care and some hospital care, despite the heterogeneity of multiple external health systems. With external funding by the Centers for Disease Control and Prevention and UC Office of the President, UCD established the first bidirectional electronic referral with its free state quitline [9], along with other electronic medical record (EMR) modifications to simplify ordering medications and referrals by physicians in the outpatient and inpatient setting. As above, the HME department has offered group classes but a barrier remains that it is not always available at all clinic sites. The need for ongoing provider training or improved clinic workflow presents some barriers for tobacco treatment, but HME has worked increasingly towards population health management with support from ambulatory care leadership. With its multidisciplinary team including pharmacists and data analysts with EMR reporting tools, the HME team has conducted proactive outreach to patients who smoke and coordinated furnishing medications. Under the UCD CCC Office of Community Outreach and Engagement, the tobacco treatment program is supported to help engage the broader catchment area’s population and partners, and the UCD CCC affiliation helps mitigate barriers while interfacing with multiple entities.

#### 3.3.2. Screening: Lung Cancer Screening as a Model

Cancer screening activities at the UCD are primarily physician-driven with reminders to primary care providers in health maintenance alerts but are not linked to tobacco assessment or treatment activities except for LCS. Leadership Engagement on tobacco treatment has been important for implementation readiness within the UCD Comprehensive LCS program, accredited in 2015 by the American College of Radiology, and other external health systems may have their own programs or contractual relationships. General thoracic surgery leads the program in conjunction with a multidisciplinary team of radiologists, pulmonologists, medical oncologists, radiation oncologists, along with the tobacco treatment team as an active member.

Available Resources and Information and Knowledge Sharing present key barriers. There are insufficient funds to have a dedicated LCS clinic, and primary care providers have competing priorities to identify eligible current or former smokers and to conduct LCS. Despite outreach and online training efforts, primary care physician referrals remain low, although the true denominator of eligible patients is unknown as detailed tobacco history in the EMR is often incomplete. The tobacco treatment program can facilitate implementation readiness through Information and Knowledge Sharing with outreach workflow and workbench reports. With ambulatory leadership support, the LCS team has piloted population outreach for LCS eligibility screening among primary care patients 55 years and older who were identified in an HME workbench report as current smokers. This outreach is being conducted first with research assistants, as sustainable workflow options through HME and/or primary care pre-visit planning nurses are being explored. There is potential for the tobacco treatment team to follow-up all patients with an LCS order who are current tobacco users. Future activities may also include the tobacco treatment team conducting LCS outreach with cancer survivors as a potential screening program pilot.

#### 3.3.3. Diagnosis: From Various Settings into Cancer Clinics

New patients to the UCD CCC with a cancer diagnosis come from a variety of internal or external settings from the hospital or primary care, where diagnostic work-up has been completed. Information and Knowledge Sharing is a barrier with the new patient referral process as smoking information is collected at the time of referral with a medical history questionnaire, but administrative staff have limited time and hard copies are scanned and not entered into the EMR. If a patient had UCD care, the EMR tobacco history section may have previously been completed by primary care clinic medical assistants or nurses or pharmacy students at the hospital; however, it was not initially part of the cancer center medical assistant workflow. This discrepancy is due in part to Leadership Engagement barriers with the cancer center clinic operations reporting historically to the hospital, and not being integrated with the rest of ambulatory operations where tobacco assessment had been prioritized in primary care. An estimated less than half of patients at the UCD CCC have primary care providers external to UCD, leaving tobacco assessment as “never assessed” in the EMR. Upon identification of this assessment gap, clinic management leadership was willing to add the assessment to the medical assistant workflow, and training was provided as an Available Resource. The next steps to improve Information and Knowledge Sharing include integrating the NCI Cancer Tobacco Use Questionnaire within the EMR for availability throughout cancer care.

#### 3.3.4. Treatment: Cancer Clinic or Hospital Services

Leadership Engagement with the NCI C3I award plus the UCD institutional commitment and subsequently with the UCD Cancer Committee infrastructure was key to identifying Available Resources and Information or Knowledge Sharing to adapt workflows for cancer care. Per the UCD CCC cancer registry abstractor who collects new cancer patient data, tobacco status was typically being documented within the cancer provider’s notes and not available in the EMR Tobacco History. However, despite the tobacco treatment services in Prevention being available to cancer patients, they were underutilized in the cancer center with a baseline pre-award rate of 4%.

With funding provided by the NCI C3I award, a cancer nurse program manager with experience in the cancer clinics and quality improvement was onboarded to help develop and integrate the tobacco treatment program into cancer care. Training on tobacco treatment in oncology for the program manager was provided for free by an external NCI cancer center, which facilitated implementation readiness. While cancer pharmacy was identified as potentially supporting a clinical pathway, multiple barriers were identified with Available Resources for physical space, staffing, and time and Information or Knowledge Sharing with using a separate electronic system that prohibited timely communication in the EMR. Available Resources also made it difficult for the HME group class to be on-site at the cancer clinics, or for clinic staff to be embedded for interventions as a tobacco treatment specialist. Only the Ear, Nose and Throat department, which had a clinic in another building, had a nurse practitioner who incorporated tobacco treatment into dedicated follow-up visits. Another Information or Knowledge Sharing barrier was that radiation oncology used a separate EMR module that did not allow easy access to referring to the tobacco treatment program. Moreover, in the hospital setting, there is a dedicated unit for patients with cancer, yet these patients may be admitted throughout the hospital on different clinical services presenting a barrier for coordinated care. Subsequent efforts to advance the implementation process is described below.

The information technology infrastructure at the health system, while mature, presented an initial Information or Knowledge Sharing barrier for data analytics. The project team requested to adapt the existing tobacco quality metric report for primary care into the cancer care setting, as population registries had not been finalized. This was complicated by the funding source on the request form being listed as the National Cancer Institute, which was viewed as a funding agency for research instead of clinical operations or quality improvement. Different teams support such workbench reports for clinical operations, quality improvement, or research activities across departments. After a discussion with the managers of three project teams, it was decided that the report writer for the original tobacco quality metric report could work to adapt the time periods and clinical departments of the report for the NCI reporting requirements.

#### 3.3.5. Survivorship: Supportive Oncology 

The Supportive Oncology and Survivorship program provides an ideal organizational home for tobacco treatment activity, offering ready integration into cancer care as well as the advantage of being embedded within larger, sustainable programs. Supportive Oncology and Survivorship encompasses an array of clinical services (e.g., psychosocial oncology, palliative care, psycho-oncology, nutrition) and educational programming, and enhanced care pathways for targeted cancer populations, including adolescents and young adults. Services are provided by a diverse, interprofessional team of clinicians, navigators, and program staff. As clinical staff have expertise in motivational interviewing and behavior change, there is a potential opportunity to develop referral pathways and in-house, in-person tobacco cessation counseling services. Leadership Engagement at the director and staff level for tobacco treatment are strong, but the administrative priority is to develop a comprehensive program and there is limited funding to hire staff dedicated for tobacco treatment. As a developing program, Available Resources are limited for staff time and Information and Knowledge Sharing capacity is limited to embed tobacco screening and treatment workflows. A “supportive care screening” instrument systematically identifies multiple sources of distress including substance use, but more discussion is needed about a clinical pathway to incorporate tobacco treatment. In facilitating implementation readiness, staff are able to make referrals and survivorship nurse navigators prepare care plans that now include tobacco cessation as a key wellness component in survivorship. The physical co-location with the tobacco treatment program nurse manager and Supportive Oncology and Survivorship make this a natural alliance for integrating tobacco treatment into future workflows.

### 3.4. Implementation Process in the Treatment Domain

The variable implementation readiness of the inner setting in the Cancer Care Continuum informed the team’s decision to select a systems approach in prioritizing reaching and servicing patients in the different domains over time. Available resources and workflows in the Prevention domain could be bridged first to the Treatment domain with Available Resources and eventually better integrated with Screening, Diagnosis, and Survivorship. Information and Knowledge Sharing was a necessary first step for providers in the Treatment domain, while workflows and available data could be identified and adapted. The NCI C3I project required tobacco assessment and treatment reports every 6 months, and monthly workbench reports for referrals allowed for rapid cycle data feedback for the project team to refine and adapt activities.

Knowledge Sharing activities were conducted about tobacco treatment in cancer and related topics at the cancer center. A cancer radiation oncologist from another NCI cancer center was invited to UCD CCC to give talks about the importance of tobacco treatment in cancer and meet with clinic staff and leadership; this was a highly valuable activity for introducing culture change. Workshops and educational symposiums on tobacco topics that include more general audiences (e.g. talking to friends and family, pets and tobacco, nursing student course on design innovation, community roundtable on flavored tobacco products, vaping and lung injury, smoking cessation research) have also been regularly hosted at the cancer center. Each activity builds the awareness of the cancer center’s commitment to support patients to quit tobacco.

Information Sharing about existing physician orders for tobacco cessation medications and referrals were conducted at meetings with staff and providers at the cancer center, but barriers to engage staff became apparent. Medical oncology and surgical oncology had providers who could be trained on existing medication and referral orders, after tobacco assessment was initiated. Pediatric oncology, while supportive, raised concerns about managing caregiver stress and guilt. Radiation therapy, challenged by staffing issues, was supportive but needed time for implementation and used a different electronic system. Chemotherapy nurses were educated to counsel patients during infusion services but did not have capacity to submit a referral order that was only for physicians. Supportive Oncology staff such as social work and nutrition services were similarly limited. A paging system was explored to contact a tobacco treatment team member, but the disruption to daily workflow would be high. Cancer pharmacy, which could potentially help furnish medications with counseling and was co-located with the cancer clinics, did not frequently utilize the same EMR system as other cancer providers and this limited any regular communication. Specialized pharmacy services for oral chemotherapy had competing priorities with managing multiple medications and side effects. HME offered a pilot group class series at the cancer center but had low referrals for patients with cancer. With the barrier of relying on physicians to submit referral orders, the cancer nurse program manager became the interim default contact for referrals.

After about a year of building knowledge, only a few champion physicians were referring to the tobacco treatment program, and clinic leadership was instrumental to develop a new workflow solution and overcome barriers. Upon data feedback with clinic leadership, it was proposed to incorporate tobacco referrals into the team-based care model where providers, nurses, and medical assistants would work in disease-site specific teams. This would help mitigate the barriers for Available Resources and Information and Knowledge Sharing by having the clinic’s medical assistants take the time to assess and obtain consent for referrals. To improve referral rates, a clinic workflow pilot was introduced with thoracic surgery, which leads the LCS program, to have medical assistants pend quitline referral orders to the physician, if they identified and consented a tobacco user during the rooming process. This modification was successful to increase referrals, but still did not include nursing or other staff who interfaced with patients. With a demonstrated workflow need, the tobacco treatment team has worked with the director of ambulatory care nursing and the medical director for the outpatient EMR to eliminate the Information and Knowledge Sharing barrier of a physician co-sign requirement for referral orders.

Within the Office of Community Outreach and Engagement, the tobacco treatment program goals have become defined to address tobacco at the patient, population, and policy levels. Workflows are being refined for ensuring that medications are also offered in conjunction with the tobacco treatment team and pharmacy services, similar to activities in the Prevention domain, that help mitigate the Available Resources barriers. The population outreach of identified tobacco users, similar to activities started in the Prevention domain, has already begun to fill care gaps, where patients have no documented tobacco assistance. Direct-to-patient outreach and population health management outside of the clinical encounter, in conjunction with the cancer center marketing staff, will help implement an opt-out approach through care coordination that facilitates Information and Knowledge Sharing activities. Policy support at the clinic workflow level and campus smoke and tobacco-free policy level help to establish a supportive environment. Over the next three years, the UCD tobacco treatment project team will discuss how teams across the Prevention and Survivorship domains can collaborate to sustain patient and population health management across the cancer care continuum.

## 4. Discussion

This study demonstrates the significant implementation challenges of integrating a sustainable tobacco treatment program at an NCI Comprehensive Cancer Center, despite the previous implementation of a mature tobacco treatment program in the broader health system. Using the Cancer Care Continuum as a systems framework and constructs of implementation readiness, the UCD CCC experience shows that Leadership Engagement is an important first step but that the complexities of a matrix cancer center need to be considered for full integration. The outer setting demonstrates different motivational drivers for the cancer center and the broader health system. The inner setting domains of the Cancer Care Continuum have varying levels of implementation readiness, especially with the subconstructs of Available Resources and Information or Knowledge Sharing. Over the two-year implementation process, the UCD CCC focused on enhancing information and knowledge sharing within the Treatment domain with the support of the Cancer Committee infrastructure, while identifying Available Resources and adapting workflows for the various cancer care service lines that intersect with the Prevention domain.

The outer setting has additional opportunities that can be explored to strengthen an institutional commitment for tobacco treatment integration. A National Academy of Sciences report on delivering high-quality cancer care states improvements are urgently needed to address a “system in crisis” [11], and translation into clinical practice is needed for patient-centered, evidence-based, and cost-conscious cancer care across the continuum [27]. The 2020 Commission on Cancer Standards was recently updated and tobacco treatment might be addressed within standards for patient care protocols, data surveillance and systems, quality improvement, community outreach, and research. Data reporting for cancer registry or clinical trials now includes tobacco and can improve evaluations of new cancer therapies by adjusting for tobacco use in analyses. The Centers for Disease Control required cancer registries to start collecting data on tobacco status as of 2012, and data collection by cancer registry abstractors is improving as with any new data requirement [10]. For clinical trials, the NCI has developed and validated the Cancer Patient Tobacco Use Questionnaire about tobacco use or exposure [28,29], which at a minimum, is recommended at registration and at end of protocol therapy. Other time points are recommended at key points before and after cancer surgery, radiation therapy, or systemic therapy; a shorter version may also be utilized for routine clinical care. Potentially, cancer providers may align their quality improvement efforts with other collaborative efforts, such as cancer care models and networks [30]. In future, tobacco treatment should be elevated to a standard for cancer care treatment, and, through required data or accreditation efforts, it may be prioritized.

The complexity of the inner setting across the Cancer Care Continuum domains highlights how interprofessional teams are necessary to engage on tobacco treatment and that population health strategies can be important. The key to the UCD CCC systems approach in program development was the philosophy that all staff have a role in supporting patients to be tobacco-free. Coordinating with the different service lines and non-physician staff caring for patients with cancer adds to the reach and integration of tobacco treatment as a standard of care, as oncologists have barriers like competing priorities to treating tobacco [31]. Such an interprofessional team can be valuable for tobacco treatment in collaborative care and may benefit from additional training [32,33]. Population health strategies can also enhance implementation readiness across the domains and care coordination at the patient level. Proactive workflows with an opt-out approach to a state quitline can be utilized for increasing reach among patients with cancer [34]. Tobacco use and its disparities can be considered within a broader socio-ecological framework across the life and cancer continuum [35]. With EMR demographic data, future targeted outreach can also be done to engage special populations that have higher tobacco use rates including Medicaid members, racial/ethnic minorities, rural populations, and sexual and gender minorities. A dedicated patient or community committee could be better integrated into ongoing efforts to adapt and refine tobacco treatment activities, as the UCD Cancer Committee does not have a patient representative and the UCD CCC Office of Community Outreach and Engagement community advisory committee and expert stakeholders review multiple cancer topics and programs. Statewide tobacco learning collaboratives may also target cancer centers to accelerate integration of tobacco treatment [25].

## 5. Conclusions

The UCD CCC findings, while they may not be generalizable to all oncology settings but mostly to NCI-designated comprehensive cancer centers in academic health systems, demonstrate the application of a conceptual framework to accelerate implementation of a sustainable tobacco treatment program across the Cancer Care Continuum. As other programs attempt to implement sustainable tobacco treatment in oncology settings, the key recommendations are to 1) identify the tobacco treatment interventions available, 2) assess the outer setting motivations of the cancer center and surrounding health systems, and 3) define the inner setting’s Implementation Readiness (Leadership Engagement, Available Resources, Information or Knowledge Sharing) across the Cancer Care Continuum. This systems approach can identify potential synergies to accelerate implementation and develop a population-based approach. Key common elements that may be shared across oncology settings include a state quitline for an adaptable intervention, cancer committees for outer/inner setting infrastructure, tobacco quality metrics for data reporting, and non-physician staff for integrated services.

The 2020 Surgeon General’s Report on Smoking Cessation concludes that smoking cessation after a cancer diagnosis can benefit overall mortality [12], which creates an imperative for all cancer centers to integrate tobacco treatment as a standard of care. At the UCD CCC, we believe that the responsibility of an NCI cancer center is to assure optimal treatment outcomes for patients with cancer and hence, tobacco cessation is essential. For us, this is not the end of our NCI C3I project, but the beginning of our emerging sustainable tobacco treatment program at the UCD CCC.

## Figures and Tables

**Table 1:** Three Subconstructs of Implementation Readiness for Tobacco Treatment with Barriers and Facilitators across the Cancer Care Continuum, UC Davis Health.

Readiness Subconstruct	Factor Addressed	Prevention	Screening	Diagnosis	Treatment	Survivorship
Leadership Engagement: Barriers	Administrative level	Variable coordination with county or state health departments	Variable coordination with external community health systems and insurance plans	Cancer clinic operations report to the hospital and not to ambulatory care operations	Cancer pharmacy has limited staffing to support furnishing and counseling for nicotine replacement medication	Commitment priorities are for providing clinical services to specific populations
	Department or clinic level	Multiple leaders across primary and specialty care services	Multiple leaders across primary and specialty care services	Multiple leaders across hospital-based teams, primary care (especially external) and oncology services	Some departments have clinics and leadership external to cancer clinics	
Leadership Engagement: Facilitators	Administrative level	Executive leadership support for program and tobacco quality metrics UCD CCC Office of Community Outreach and Engagement support	Leadership support (ambulatory care, population health) for Lung Cancer Screening (LCS) program		Cancer Center Director, Physician-in-Chief, and Executive Director of oncology services support program activities	
	Department or clinic level	Ambulatory Care Nursing and Medical Directors support workflow/IT changes	LCS committee chair includes tobacco treatment program staff	Hospital-based pharmacy faculty incorporate student learners to assist some patients for tobacco treatment	Cancer Committee adopted tobacco treatment quality improvement as a programmatic goal Clinic supervisors engaged with supporting program	Supportive Oncology and Survivorship Director includes tobacco treatment program staff
Available Resources: Barriers	Money	Insufficient funding for TTS in every clinic	Insufficient funds for dedicated nurse practitioner to manage a LCS clinic that could include tobacco treatment	External primary care clinics may not have funds for TTS	Insufficient funding for TTS in every department or clinic	Limited funding for hiring additional staff for substance use or tobacco treatment while addressing psychological distress
	Training or education	Tobacco treatment not part of annual provider training	Cancer screening not part of annual provider training	External primary care clinics referring new cancer patients may not have tobacco treatment workflow		
	Physical space	HME group class rotates across different clinic sites every month.	LCS has only 1-2 clinic sites for PCP referrals		Limited cancer clinic space for classes	Limited space for additional staff
	Time		Primary care has competing priorities to conduct LCS	Staff processing new patient referrals have limited time	Limited cancer clinic staff time for interventions	Limited time for Supportive Oncology and Survivorship staff for interventions
Available Resources: Facilitators	Money	Free state quitline services	Ambulatory care support for LCS program		Health system leadership commits resources to sustain tobacco treatment program	
	Training or education	Staff training for tobacco treatment in oncology	Online provider training video for LCS referrals	Staff training for tobacco treatment in oncology	Medical assistant training on assessment	
	Physical space	HME conducts online group class			Cancer pharmacy in cancer clinic building	Nurse program manager co-located with Supportive Oncology and Survivorship
	Time				Public Affairs and marketing staff promote program	
Access to Information and Knowledge: Barriers	Workflow in clinical setting	Communication gap between rooming assessment and provider social history	Tobacco treatment not mandated in cancer screening, except for LCS External referrals for LCS depends on contractual arrangements	New patient referral workflow processing paperwork does not make referrals to tobacco treatment Medical assistants initially not required to assess tobacco status	Providers document tobacco in notes instead of the EMR Tobacco History Data analytics challenging with IT report teams for research, clinical operations, quality improvement Hospital cancer patients on different clinical services	No routine review of tobacco assessment or referrals with patient outreach Supportive care screening questionnaire includes self-reported substance use but not tobacco
	Information technology	Referring health systems may not have tobacco treatment tracking and referral systems	EMR tobacco history section challenging to identify LCS eligibility accurately	New cancer patient questionnaire not entered into EMR Tobacco History	Cancer pharmacy and radiation oncology utilize different electronic systems	Delays in production for population registries
	Training or education	Brief provider/clinic staff meetings	No ongoing training		Brief provider/clinic meetings or huddles	
Access to Information and Knowledge:Facilitators	Workflow in clinical setting	Primary care workflow for tobacco treatment. HME uses workbench reports for outreach	Tobacco treatment program collaborating on outreach to eligible patients	Hospital teams or UCD primary care may already assess or refer	Medical assistants assess and refer patients. Cancer pharmacy affiliated with outpatient pharmacy	Interprofessional team helps to refer. Navigators added tobacco to survivorship care plans
	Information technology	EMR Health Maintenance Alert, tobacco treatment orders, tobacco registry	EMR Health Maintenance alert for LCS has link to order		Cancer Patient Tobacco Use Questionnaire; tobacco registry for oncology patients (pending)	
	Training or education		Pre-Visit Planners in UCD primary care clinics		Medical assistants and nurses trained on referrals	Supportive oncology team trained on referrals

UCD CCC = UC Davis Comprehensive Cancer Center, LCS = lung cancer screening, EMR = electronic medical record, HME = Health Management Education, TTS = tobacco treatment specialist.
